# The Impact of Perioperative Multimodal Pain Management on Postoperative Outcomes in Patients (Aged 75 and Older) Undergoing Short-Segment Lumbar Fusion Surgery

**DOI:** 10.1155/2022/9052246

**Published:** 2022-02-27

**Authors:** Shuaikang Wang, Tongtong Zhang, Peng Wang, Xiangyu Li, Chao Kong, Wenzhi Sun, Shibao Lu

**Affiliations:** ^1^Department of Orthopedics, Xuanwu Hospital, Capital Medical University, No. 45 Changchun Street, Xicheng District, Beijing, China; ^2^National Clinical Research Center for Geriatric Diseases, Beijing, China; ^3^Department of Orthopedics, Chuiyangliu Hospital Affiliated to Tsinghua University, Beijing 100078, China

## Abstract

**Background:**

Due to the presence of multimorbidity and polypharmacy, patients aged 75 and older are at a higher risk for postoperative adverse events after lumbar fusion surgery. More effective enhanced recovery pathway is needed for these patients. Pain control is a crucial part of perioperative management. The objective of this study is to determine the impact of multimodal pain management on pain control, opioid consumption, and other outcomes.

**Methods:**

This is a retrospective review of a prospective collected database. Consecutive patients who underwent elective posterior lumbar fusion surgery (PLF) from October 2017 to April 2021 in our hospital were reviewed. Perioperative multimodal pain management (PMPM) group (from January 2019 to April 2021) in which patients received multimodal analgesia was case-matched to the control group (from October 2017 to December 2018) in which patients were treated under the conventional patient-controlled analgesia (PCA) method. Postoperative visual analogue scale (VAS), opioid consumption, complications within 3 months, and other outcomes were collected and compared between groups.

**Results:**

A total of 122 consecutive patients (aged 75 and older) were included in the PMPM group and compared with previous 122 patients. The PMPM group had a lower maximal VAS score (3.0 ± 1.7 vs. 3.7 ± 2.0, *p* < 0.001) and frequency of additional opioid consumption (6.6% vs. 19.7%, *p*=0.001) on POD3 than the control group. The rates of postoperative complications were lower in the PMPM group compared with the control group (25% vs. 49%, *p*=0.006) during a 3-month follow-up period.

**Conclusions:**

This study demonstrates that the PMPM protocol is effective in pain control and reducing additional opioid consumption when compared with conventional analgesia, even for patients aged 75 and older. Moreover, these improvements occur with a lower incidence of postoperative complications within three months after PLF surgery.

## 1. Introduction

With rapid population aging in many countries, the incidence of lumbar degenerative disease is gradually increasing and seriously deteriorating the quality of life of patients [1, 2]. Short-segment (one- or two-level) posterior lumbar interbody fusion (PLF) surgery with or without depression is an important way to treat lumbar degenerative diseases such as lumbar disk herniation (LDH), lumbar spinal stenosis, and lumbar spondylolisthesis [3]. Age is a risk factor for increased incidence of postoperative complications after PLF; however, age is not associated with worse patient-reported outcomes [4]. Due to the presence of multimorbidity and polypharmacy, patients with age 75 and older are at a higher risk for postoperative adverse events, which increases the costs of hospitalization [5]. Efforts are needed to accelerate recovery after surgery and improve these patient's clinical outcomes and experience.

Poor pain control is associated with patients' dissatisfaction [6], postoperative complications [7], and excessive opioid consumption [8]. Patient-controlled analgesia (PCA) and perioperative multimodal pain management (PMPM) (also known as multimodal analgesia) relive unnecessary suffering after fusion surgery [9]. PCA is a conventional method that allows the patients to self-administer intravenous opioid medication to control pain [10]. Perioperative opioid use was associated with gastrointestinal complications [8, 11], more extended hospital stays [12], and long-term opioid use [13] after surgery. Multimodal pain management involves a combination of acetaminophen, pregabalin, gabapentin, cyclooxygenase-2 (COX-2) inhibitors, steroids, and neuraxial anesthesia with different mechanisms of action to reduce the use of opioids and the incidence of opioid-related adverse events [14]. Since Kehlet et al. [15] proposed the effects of multimodal analgesia, multimodal pain management had been implemented in animal studies and perioperative pain control. Durand et al. [16] found that multimodal analgesia was more effective in long-term pain management following castration in sheep. Coutens et al. [17] also found that the combination of morphine with ketamine or ketoprofen produced antinociceptive responses in animals with severe nociceptive acute pain induced by a closed tibial fracture.

At present, despite great advances in medicine and infusion devices in recent decades, opioids remain the primary drug to achieve adequate pain control. Given the side effects of opioids, effective multimodal medication regimens are needed to reduce postoperative opioid use and improve outcomes without increasing pain levels in older patients. Previous studies had demonstrated the associations between PMPM and outcomes including cost reduction, less morphine consumption, shorter length of hospitalization, and lower complications rates in various patient cohorts with an average age range of 50–70 years [9, 18–21]. However, few studies reported PMPM implementation in older patients undergoing lumbar fusion surgery. To our knowledge, this is the first report on the role of PMPM in patients aged 75 and older. Our primary aim was to compare the efficiency of our multimodal pain management program (i.e., reducing postoperative pain levels and opioid use during hospitalization) to a traditional pain management method, and the secondary aim was to evaluate the impact of multimodal pain management on length of hospital stays (LOS), postoperative complications, and readmission within three months in patients (aged 75 and older) undergoing short-segment lumbar fusion surgery.

## 2. Materials and Methods

This was a single-center retrospective study. We reviewed consecutive patients who underwent elective posterior lumbar fusion surgery for degenerative lumbar spinal stenosis, lumbar disc herniation, and lumbar spondylolisthesis. The same surgical team performed surgery from October 2017 to April 2021 in our hospital, and data from the electric medical records' system and prescription records were collected. Approval was obtained from the ethics committee of our hospital (permit data 2018.4.3; no. 2018086).

### 2.1. Inclusion and Exclusion Criteria

The inclusion criteria were as follows: (1) age 75 and older; (2) short-segment fusion surgery for lumbar degenerative disease. The exclusion criteria were as follows: (1) revision surgery; (2) emergency surgery; (3) lumbar tuberculosis and tumor; (4) incomplete perioperative clinical data.

### 2.2. Surgical Technique

We reviewed all patients who underwent depression with standard posterior lumbar fusion. Under general anesthesia, the patient was placed on the operating table in a prone position. The surgical approach was chosen depending on the planned range of decompression. A midline incision was made for all patients. For patients undergoing the traditional approach, the erector spinae muscles were separated from lumbar bony elements to expose the lamina and facet joints and transverse as needed for the levels that must be visualized. For patients undergoing open-Wiltse approach, only the plane between the multifidus and longissimus muscles was exposed by blunt dissection. The vertebral pedicle screws of surgical segments were implanted according to preoperative radiography and intraoperative fluoroscopy. The nerve roots were decompressed by hemilaminectomy or laminectomy according to the preoperative lumbar symptoms and radicular symptoms and MRI. After removal of the intervertebral disc, the bone graft was placed at the anterior part of the intervertebral space, the cage filled with the bone graft was also implanted into the intervertebral space, and at last, the remaining part of autogenous bone grafts from the decompression laminectomy was placed in the bone bed. Once the position and direction of implants were satisfactory, the wound was flushed, and the drainage tube was placed, incision was sutured layer by layer.

### 2.3. Perioperative Pain Management

An enhanced recovery after surgery (ERAS) protocol was applied in our institute from January 2019 with the multimodal analgesia as the only pain management method, and the patients were divided into a control group (from October 2017 to December 2018) in which patients were treated with the conventional PCA method and a case-matched PMPM group (from January 2019 to April 2021). Intraoperatively, both groups received general anesthesia with intravenous propofol and remifentanil according to patients' weight and operation time. In the PMPM group, all patients were given 150 mg of pregabalin 2 h before surgery. A mixture of 10 ml 2% lidocaine and 10 ml 1% ropivacaine was infiltrated around the surgical incision before incision and after skin closure. All patients received an intravenous cyclooxygenase-2 (COX-2) infusion on postoperative day 0 (POD0), POD1, and POD2. In the PMPM group, pain medications were prescribed according to the World Health Organization's (WHO) three-step analgesic ladder protocol. Oral or intravenous drugs were used to improve perioperative analgesia with the nonopioid drug as the first choice (which differed from the control group). In the control group, pain medications were prescribed according to the experience of the attending physicians, and PCA (containing sufentanil and other agents in 100 mL saline) was used for anesthesia on POD0, POD1, and POD2 (Table 1).

### 2.4. Outcome Measure

We extracted age, gender, body mass index (BMI), comorbidities, primary diagnosis, American Society of Anesthesiologists score (ASA score), and visual analogue scale (VAS) of the leg and lower back. Operation-related variables from the electronic medical records' system and perioperative opioid prescription information from the prescription monitoring program were collected. The primary outcomes were additional oral opioids' doses and postoperative maximal VAS score on postoperative days 1, 2, and 3. The secondary outcomes were the day of first ambulation and postoperative complications within three months of surgery, postoperative LOS, and readmissions within 3 months. Two independent researchers analyzed all data.

### 2.5. Statistical Analysis

All continuous variables (e.g., age) were presented as mean ± standard deviation and analyzed using the two-tailed Student's *t*-test and one-way ANOVA. For nonnormally distributed data, data conversion or the Mann–Whitney test was used. Qualitative variables (such as gender) were represented as frequency (percentages) and analyzed using Fisher's exact or chi-square tests. SPSS software (version 22.0; SPSS Inc., Chicago, IL, USA) was used for statistical analysis. Significance was set at *p* < 0.05.

## 3. Results

A total of 122 consecutive patients in the PMPM group received multimodal analgesia protocol at our institute. Baseline data for these patients were compared to the previous 122 consecutive patients (from October 2017 to December 2018), and no differences were observed in age, gender, BMI, and fused levels; therefore, further matching was not attempted. In the PMPM group, 62.3% of patients were female, with an average age of 77.9 years. The average age was 77.9 years in the control group, and 59.0% were female. No significant differences were observed between two groups in ASA scores or surgery-related variables (Table 2).

**Figure 1:**
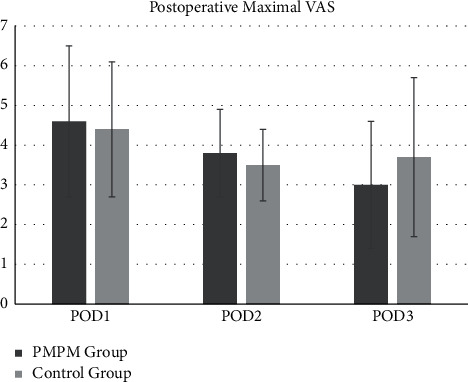
The maximal VAS score on POD1, POD2, and POD3 of patients in the PMPM group and control group.

**Figure 2:**
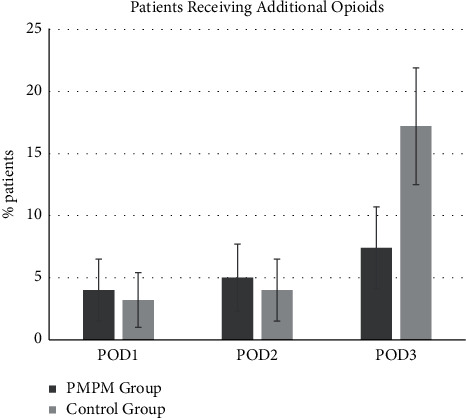
Percentages of patients receiving additional oral opioids on POD1, POD2, and POD3 for the PMPM group and control group.

The pain level was defined as the maximal VAS score in the current study. The VAS scores were similar on POD1 between groups and were higher in the PMPM group than in the control group on POD2 (however, without reaching statistical significance). The maximal VAS score was significantly lower on POD3 in the PMPM group than the control group (3.0 ± 1.7 vs. 3.7 ± 2.0, *p* < 0.001) (Figure 1). No significant differences were observed in the frequency of additional oral opioid prescriptions between the two groups on POD1 and POD2; however, the frequency and percentages were significantly lower in the PMPM group than in the control group on POD3 (6.6% vs. 19.7%, *p*=0.001) (Figure 2), and total oral opioid consumption was lower in the PMPM group (213 mg vs. 655 mg) (Table 3).

The rates of postoperative complications were lower in the PMPM group than the control group (25% vs. 49%, *p*=0.006) during the 3-month follow-up. The most common complications in both groups were constipation and hypoalbuminemia. The PMPM group had a lower incidence than the control group for constipation (18% vs. 28.7%, *p*=0.049) and hypoalbuminemia (13% vs. 38%, *P*=0.012); however, there were no differences in other complications including surgical site infection (SSI) and urine retention. The rates of 3-month readmission and transferring to rehabilitation were similar between the groups, with shorter postoperative LOS (7.7 ± 3.9 vs. 9.0 ± 4.1, *p*=0.013) and frequency of extended LOS (28% vs. 42%, *p*=0.023) in the PMPM group. The average time of first bedside ambulation was 1.7 days in the PMPM group and 4.1 days in the control group after surgery (Table 4).

## 4. Discussion

Due to the presence of more significant risks of frailty and comorbidity, the incidences of postoperative complications and mortality are higher in patients aged 75 and older; for these reasons, careful perioperative management protocol of these patients is needed [22]. Postoperative pain control is an essential component of ERAS. Inadequate pain control is detrimental to early mobilization and recovery and is associated with increased LOS, costs of hospitalization, and incidence of postoperative complications [23, 24]. Although many nonopioid analgesics were prescribed for pain management after orthopedic surgery, the use of opioids continues to increase. Opioid overdoses are associated with a higher risk of death and postoperative complications, including constipation, nausea, vomiting, and urinary retention [8]. The minimization of postoperative opioid consumption relies on the comprehensive analgesia protocol and is critical in the context of the opioid epidemic. Traditional analgesia methods include nurse-controlled analgesia and PCA. PCA is effective for pain control; however, it increases the use of opioid and opioid-related side effects [10]. In the current study, we hoped to evaluate the effects of the multimodal analgesia pathway on pain control and other outcomes in older patients in China.

Multimodal analgesia is an alternative to PCA and is based on concurrent use of primary nonopioid agents. Nonsteroidal anti-inflammatory drugs (NSAIDs) are effective analgesics for musculoskeletal pain control; they inhibit cyclooxygenase (COX) isozymes and decrease prostaglandin generation. Acetaminophen produces an analgesic effect through peripheral and central COX inhibition like NSAIDs. Jirarattanaphochai and Jung [25] reviewed 17 randomized controlled trials and found that the addition of NSAIDs to opioid analgesics provided better pain control than opioid analgesics alone. However, a previous study had shown that NSAIDs had dose-dependent and duration-dependent effects on fusion rates, and high-dose COX inhibitors decreased fusion rates [26]. As structural analogues of gamma-aminobutyric acid, gabapentin and pregabalin could relieve acute and chronic neuropathic pain through reducing neuronal excitability. A systematic review and meta-analysis performed by Hurley et al. showed that patients receiving preoperative pregabalin had a significant decrease in postoperative neuropathic pain significantly [27]. Combining these drugs with different mechanisms of action has synergistic analgesic effects on postoperative pain and reduces the dose of single-agent doses.

PMPM is a comprehensive protocol including multiple analgesic strategies. Schotanus et al. [28] performed a randomized controlled trial and found that single-shot local infiltration analgesia with ropivacaine alone resulted in clinical acceptable adequate pain control in patients undergoing total knee arthroplasty. In the present study, preemptive analgesia and local infiltration analgesia were applied in patients of the PMPM group. Our PMPM protocol improved pain control on postoperative day 3, which was consistent with previous studies. Rajpal et al. [19] reported that preventative multimodal analgesia improved pain control on all four postoperative days in patients undergoing lumbar fusion surgery, and Choi et al. [9] found that multimodal analgesia reduced additional opioid use on postoperative day 2 without increasing pain levels in patients with one- or two-level posterior lumbar fusion surgery compared to a PCA group. In our study, more physical activity might contribute to the slightly increased VAS score and additional opioids' prescription on POD3 in the PMPM group; the analgesic pump would be turned off on POD3, which might contribute to a significantly increased VAS score in the control group. A previous study reported that opioid requirements were lower in the older patients but were associated with more adverse events [29].

In the present study, we identified that the PMPM group had less use of opioids without increasing the level of postoperative pain and incidence of severe complications in patients (75 years or older). Compared with the control group, the incidences of postoperative complications in the PMPM group were lower (especially opioid-related complications, such as postoperative constipation and nausea/vomiting). The use of opioid has a suppressive effect on the respiratory center and provoked nausea and vomiting by activation of central chemoreceptors. Poor pain control is also associated with postoperative complications [7]. The reduction of opioid use and adequate pain control may contribute to a low incidence of opioid-related complications [8, 14]. There was no difference between the groups in deep venous thrombosis, urinary tract infections, and wound infections. A retrospective study conducted by Pirkle et al. [30] found that chronic opioid use was associated with surgical wound infections; however, the underlying mechanisms for this observation remain unclear. The present study found that the multimodal analgesia pathway was associated with less postoperative hypoproteinemia. The reasons for this result might be as follows: firstly, patients in the PMPM group had a lower risk for gastrointestinal complications after surgery, and secondly, improved pain control may make patients feel more at ease than the control group. Our PMPM program achieved the goal of early mobilization without increasing postoperative pain levels. A retrospective study found that early ambulation was associated with decreased postoperative adverse events [31]; in our study, most patients were more likely to ambulate on POD1 in the PMPM group and on POD4 in the control group. Previous studies demonstrated an association between opioid agonists and serious postoperative complications following orthopedic procedures [32, 33]. The safety of PMPM had been validated in other studies; the rates of respiratory depression, acute renal failure, and central nervous system complications were not higher in the PMPM group than in the non-PMPM group after spinal surgery and total knee arthroplasty [21, 34]. In the present study, the rates of postoperative delirium, acute myocardial infarction, and acute cerebral infarction were similar between groups.

Because of the higher risk for extending postoperative LOS in patients aged 75 and older, the average LOS of patients in our study was more prolonged than shown in other studies; however, patients in the PMPM group had a shorter postoperative LOS. Tank et al. [12] found that opioid dependence was associated with prolonged LOS following lumbar fusion. Our multimodal analgesia protocol combined opioid and nonopioid analgesic mechanisms to achieve additive or synergistic effects on pain control. ERAS pain management protocols emphasize a multidisciplinary and comprehensive approach across the operative episode to enhance postoperative recovery and minimize opioids' consumption [14]. A previous study showed that ERAS reduced LOS and hospital costs significantly in older adults [25]; however, little attention has been paid to the contribution that ERAS and multimodal analgesia might make to achieving the same goals considerably in older patients (aged 75 or older). In the present study, we identified that PMPM resulted in clinical acceptable adequate pain control in patients undergoing short-segment fusion surgery with less opioids' consumption, which contributed to maximization of early mobilization and recovery in older patients.

Our PMPM strategy included preemptive analgesia and multimodal analgesia and ensured that the nonopioid agent was preferentially used for postoperative pain control according to the three-step analgesic ladder protocol. A randomized placebo-controlled study conducted by Fujita et al. [36] showed that administration of 150 mg of pregabalin before spine surgery decreased morphine consumption and postoperative pain intensity, but Trung Kien et al. [37] found that preoperative pregabalin combined with celecoxib orally had a good preemptive analgesic effect in lumbar spine surgery. Vasigh et al. [38] also showed that the effect of gabapentin plus celecoxib on pain was better than gabapentin alone after laminectomy. Further research should attempt to establish a better combination of preemptive analgesia and nonopioid analgesia based on recent advancements in analgesics and synergistic effects of various narcotics.

There are several limitations to the present study. First, this was not a randomized controlled study and was subject to inherent limitations associated with retrospective analyses; nevertheless, it is unethical to perform a randomized controlled study, given that opioids have been proven to be correlated with numerous adverse events. Second, only the impact of multimodal analgesia on pain levels and opioid use on POD1, POD2, and POD3 were evaluated. The VAS scores or opioid prescription doses were not acquired after discharge. Longer follow-up is needed to determine the long-term effects of the PMPM protocol. The ways of pedicle screw implantation and the procedures of surgical decompression have an impact on postoperative lower back pain; however, we did not have a detailed record of surgical approach of each individual. Despite these limitations, our retrospective review and analysis of a prospectively collected database was the first to evaluate the effect of multimodal analgesia on patients aged 75 years and older.

## 5. Conclusions

This study demonstrates that the PMPM protocol is effective in pain control and reducing additional opioid consumption when compared with conventional analgesia, even for patients with age 75 and older, and these improvements occur with a lower incidence of postoperative complications within three months after PLF surgery. The implementation of multimodal analgesia combined with nonopioid analgesia could be recommended for accelerating recovery after fusion surgery. Further research should attempt to establish better pain management protocol-based recent advancements in analgesics and synergistic effect of different narcotic drugs.

## Figures and Tables

**Table 1:** Two different perioperative pain management protocols.

	Control group	PMPM group	Time
Preoperatively	No intervention	Step one: acetaminophen and/or NSAIDs and/or gabapentin, PO	After admission, PRN
		Step two: opioids, PO	PRN

Intraoperatively	—	150 mg of pregabalin, PO	2 h before surgery
	Propofol, IV	Propofol, IV	During surgery
	Sufentanil, IV	Sufentanil, IV	During surgery
	—	A mixture of 10 ml 2% lidocaine and 10 ml 1% ropivacaine, local anesthesia	Before incision and after skin closure

Postoperatively	PCA	Cyclooxygenase-2 (COX-2) inhibitors, IV	Day 0–day 2
	No intervention	Step one: acetaminophen and/or NSAIDs and/or gabapentin, PO	PRN
		Cyclooxygenase-2 (COX-2) inhibitors, IV	PRN
		Step two: opioids, PO	PRN

PMPM: perioperative multimodal pain management; IV: intravenous; NSAIDs: nonsteroidal anti‐inflammatory drugs; PO: peros (oral); PRN: as required.

**Table 2:** Baseline characteristics of patients in the two groups.

Variable	PMPM group (*n* = 122)	Control group (*n* = 122)	*p* value
Female, *n* (%)	76 (62.3)	72 (59.0)	*p*=0.600
Age (yr)	77.9 (74.0–81.8)	78.7 (74.8–82.6)	*p*=0.230
Height (cm)	161 (153–169)	161 (153–169)	*p*=0.075
Weight (kg)	65.1 (54.8–75.4)	64.8 (54.1–75.5)	*p*=0.896
BMI (kg/m^2^)	25.1 (21.4–28.8)	24.9 (21.3–28.5)	*p*=0.723

Comorbidities, *n* (%)			
Hypertension	86 (70)	82 (67)	*p*=0.580
Coronary heart disease	30 (25)	32 (26)	*p*=0.769
Diabetes disease	41 (33)	32 (26)	*p*=0.208
Mental disease	2 (2)	4 (3)	*p*=0.320
Digestive disease	8 (7)	7 (6)	*p*=0.790
Old cerebral infarction	14 (11)	8 (7)	*p*=0.180
Pulmonary disease	4 (3)	6 (5)	*p*=0.518
Osteoporosis	17 (14)	18 (15)	*p*=0.855
Preoperative opioid	7 (6)	9 (7)	*p*=0.605

Diagnosis			*p*=0.900
LSS	64 (52.4%)	63 (51.6%)	
LDH	39 (32.0%)	40 (32.8%)	
Lumbar spondylolisthesis	19 (15.6%)	19 (15.6%)	

VAS (lower back)	5.3 (3.2–7.4)	5.6 (3.7–7.5)	*p*=0.485
VAS (leg)	7.3 (5.9–8.7)	7.2 (5.7–8.7)	*p*=0.718
ODI	60.0 (46.6–73.4)	58.3 (44.8–71.8)	*p*=0.543

Procedure-related			
Fusion level			*p*=0.433
1	52 (42.6%)	46 (37.8%)	
2	70 (57.4%)	76 (62.2%)	
Operative time (min)	190.7 (131.9–249.5)	192.6 (145.2–240.0)	*p*=0.068
EBL (ml)	240.9 (68.2–412.0)	279.0 (115.1–443.0)	*p*=0.549

BMI: body mass index; LSS: lumbar spine stenosis; LDH: lumbar disc herniation; VAS: visual analogue scale; ODI: Oswestry Disability Index; EBL: estimated blood loss.

**Table 3:** Postoperative pain level and opioid consumption.

	PMPM group (*n* = 122)	Control group (*n* = 122)	*p* value
Maximal VAS score			
POD1	4.7 (2.8–6.6)	4.6 (2.8–6.4)	*p*=0.690
POD2	3.9 (2.8–5.0)	3.6 (2.6–4.6)	*p*=0.149
POD3	3.0 (1.2–4.6)	3.7 (1.7–5.7)	*p*=0.001^*∗*^

Additional opioid consumption, *n* (%)			
POD1	6 (4.9)	4 (3.3)	*p*=0.518
POD2	6 (4.9)	5 (4.1)	*p*=0.758
POD3	8 (6.6)	24 (19.7)	*p*=0.001^*∗*^
Total oral opioid consumption (mg)	213	655	

VAS: visual analogue scale; POD1: postoperative day 1; POD2: postoperative day 2; POD3: postoperative day 3; ^*∗*^*P* < 0.05.

**Table 4:** Other outcomes of the two groups.

	PMPM group (*n* = 122)	Control group (*n* = 122)	*p* value
Postoperative LOS	7.7 (3.8–11.6)	9.0 (4.9–13.1)	*p*=0.013^*∗*^
Extended LOS, *n* (%)	35 (28)	52 (42)	*p*=0.023^*∗*^
The day of first ambulation	1.7 (0.8–2.7)	4.1 (2.4–5.8)	*p*=0.001^*∗*^

Complications	30 (25%)	60 (49%)	*p*=0.006^*∗*^
Cardiovascular disease	1 (1%)	2 (2%)	*p*=0.561^*∗*^
Acute cerebral infarction	0	0	
Delirium	1 (1%)	2 (2%)	*p*=0.561
SSI	5 (4%)	8 (7%)	*p*=0.392
Pneumonia	2 (2%)	1 (1%)	*p*=0.561
Hematoma	1 (1%)	2 (2%)	*p*=0.561
DVT	3 (2%)	3 (2%)	*p*=0.100
Urinary tract infection	2 (2%)	3 (3%)	*p*=0.006
Nausea/vomiting	6 (4.9%)	15 (12.3%)	*p*=0.006
Retention of urine	1 (1%)	4 (3%)	*p*=0.175
Constipation	22 (18%)	35 (28.7%)	*p*=0.049^*∗*^
Hypoalbuminemia	28 (13%)	46 (38%)	*p*=0.012^*∗*^

The rate of readmission, *n* (%)	2 (2%)	7 (6%)	*p*=0.089
Transfer to rehabilitation center, *n* (%)	2 (2)	6 (5)	*p*=0.150

LOS: length of stay; SSI: surgical site infection; DVT: deep vein thrombosis; ^*∗*^*p* < 0.05.

## Data Availability

The underlying data supporting the results of this study could be obtained by contacting the corresponding author.
